# Mitochondrial oxidative stress in aging and healthspan

**DOI:** 10.1186/2046-2395-3-6

**Published:** 2014-05-01

**Authors:** Dao-Fu Dai, Ying Ann Chiao, David J Marcinek, Hazel H Szeto, Peter S Rabinovitch

**Affiliations:** 1 Department of Pathology, University of Washington, 1959 Pacific Ave NE, HSB-K081, Seattle, WA 98195, USA; 2 Department of Radiology, University of Washington, Seattle, WA, USA; 3 Department of Pharmacology, Weill Cornell Medical College, New York, NY, USA

**Keywords:** Mitochondria, Oxidative stress, Aging, Healthspan

## Abstract

The free radical theory of aging proposes that reactive oxygen species (ROS)-induced accumulation of damage to cellular macromolecules is a primary driving force of aging and a major determinant of lifespan. Although this theory is one of the most popular explanations for the cause of aging, several experimental rodent models of antioxidant manipulation have failed to affect lifespan. Moreover, antioxidant supplementation clinical trials have been largely disappointing. The mitochondrial theory of aging specifies more particularly that mitochondria are both the primary sources of ROS and the primary targets of ROS damage. In addition to effects on lifespan and aging, mitochondrial ROS have been shown to play a central role in healthspan of many vital organ systems. In this article we review the evidence supporting the role of mitochondrial oxidative stress, mitochondrial damage and dysfunction in aging and healthspan, including cardiac aging, age-dependent cardiovascular diseases, skeletal muscle aging, neurodegenerative diseases, insulin resistance and diabetes as well as age-related cancers. The crosstalk of mitochondrial ROS, redox, and other cellular signaling is briefly presented. Potential therapeutic strategies to improve mitochondrial function in aging and healthspan are reviewed, with a focus on mitochondrial protective drugs, such as the mitochondrial antioxidants MitoQ, SkQ1, and the mitochondrial protective peptide SS-31.

## Introduction

**Figure 1:**
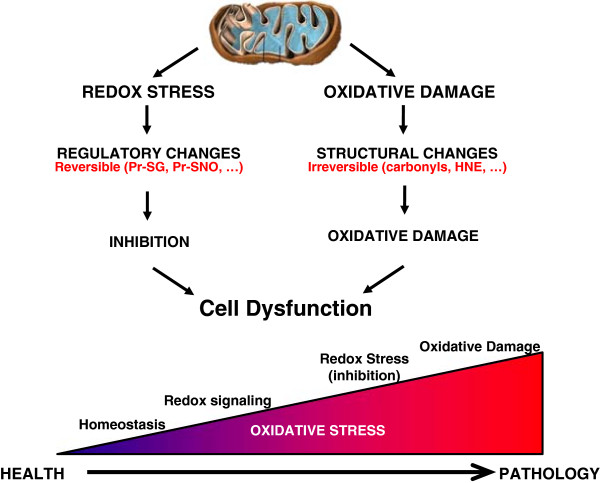
**Illustration of the continuum of oxidative stress in health and pathology.** The redox stress pathway emphasizes the signaling role of oxidative stress and focuses on reversible regulation and depends on the interaction between cellular components and the redox environment of the cell. In contrast, prolonged or high oxidative stress leads to structural changes in proteins, lipids, and DNA that are generally more irreversible. These represent two points along the continuum of how oxidative stress may contribute to aging phenotypes. Modified from Marcinek and Siegel [120].

Denham Harman first proposed the free radical theory of aging in 1956, suggesting that free radical-induced accumulation of damage to cellular macromolecules is a primary driving force of aging and a major determinant of lifespan [1]. This theory, however, is a highly simplified view of the role of reactive oxygen species (ROS) in the biology of aging. There are a number of sources of intracellular ROS in mammals, including NADPH oxidases (NOX), mitochondria, xanthine oxidase, monoamine oxidase, and nitric oxide synthase. The term ROS itself, encompasses numerous species that range from highly reactive (OH^.^) to longer-lived and membrane permeant (H2O2). Under normal conditions, ROS are maintained at the physiological levels by several endogenous antioxidant systems, including superoxide dismutatase (SOD), catalase, glutathione peroxidases, and glutathione reductase (GR). Other antioxidant systems involving thiol-disulphide oxidoreductase systems include the cytosolic proteins thioredoxin (TRX) and glutaredoxin (GRX). These antioxidant systems are complex, located in different cellular compartments and are often redundant or complementary in various conditions. Physiological levels of ROS interact with redox state and play a role in mediating cell signaling, while pathological levels of ROS can result in oxidative damage to cellular components and activate several cell death pathways (Figure 1). The close interrelationship of redox balance to oxidative stress has in recent years become a more prominent aspect of the free radical theory of aging and has been the subject of several reviews [2-4].

Based on the free radical theory of aging, several scientists have attempted to increase lifespan by genetic manipulation of antioxidant system components, however, the results have generally been conflicting. In *Caenorhabditis elegans*, single or double SOD mutants have a normal lifespan, while mitochondrial SOD2 mutants (single or double with cytoplasmic SOD1) increased lifespan [5]. In *Drosophila melanogaster* early results were confounded by uncontrolled genetic background effects. Later analyses suggested that over-expression of catalase, SOD1, or SOD2 using the genes’ native promoters does not increase life span [6,7], but that when tissue-specific or conditional transgenic overexpression systems were used, elevated SOD2 did result in substantial life span increases [8]. It has also been suggested that the largest lifespan extensions were seen in backgrounds with shorter lifespan or under redox stress [3]. In transgenic mice, the overexpression of endogenous antioxidants, including CuZnSOD (SOD1, cytoplasmic), MnSOD (SOD2, mitochondrial), catalase, or combination of CuZnSOD/catalase and CuZnSOD/MnSOD failed to extend mouse lifespan [9-11]. While SOD1 knockout mice exhibit 30% shorter lifespan, the fact that their major cause of death is hepatocellular carcinoma and the absence of lifespan reduction in SOD1 heterozygous mice suggest the shorten lifespan in SOD1 knockout may not be due to accelerated aging. While complete deletion of SOD2 cause neonatal death, SOD2 heterozygous mice and SOD3 both shown normal lifespan. However, it is notable that in many disease models or when under environmental stress, the same transgenic overexpression mice may be healthier than their wild-type counterparts, and the converse for antioxidant under-expressing mice (reviewed by [12]).

Several clinical trials using antioxidant supplementation in various study populations have been performed during the last three decades and the results are often equivocal or conflicting. Meta-analyses of large numbers of individual reports are often required to reach conclusions, however these too vary. A large scale analysis of 68 randomized trials including 232,606 participants from general population or patients with heterogeneous diseases have reported no effect of antioxidant supplements on overall mortality, or even a significant increase in mortality in subjects receiving beta carotene, vitamin A, and vitamin E [13]. A recent widely cited meta-analysis including 50 randomized controlled trials with 294,478 participants showed no evidence to support the use of vitamin and antioxidant supplements for prevention of cardiovascular diseases [14]. In contrast, a recent meta-analysis of seven studies on the risk of Alzheimer’s disease showed that dietary intakes of vitamin E, vitamin C, and beta carotene can lower the risk of AD [15]. In spite of extensive study it remains clear that there is no consensus and/or those effects are disease-dependent.

## Review

### Mitochondrial free radical theory of aging

**Figure 2:**
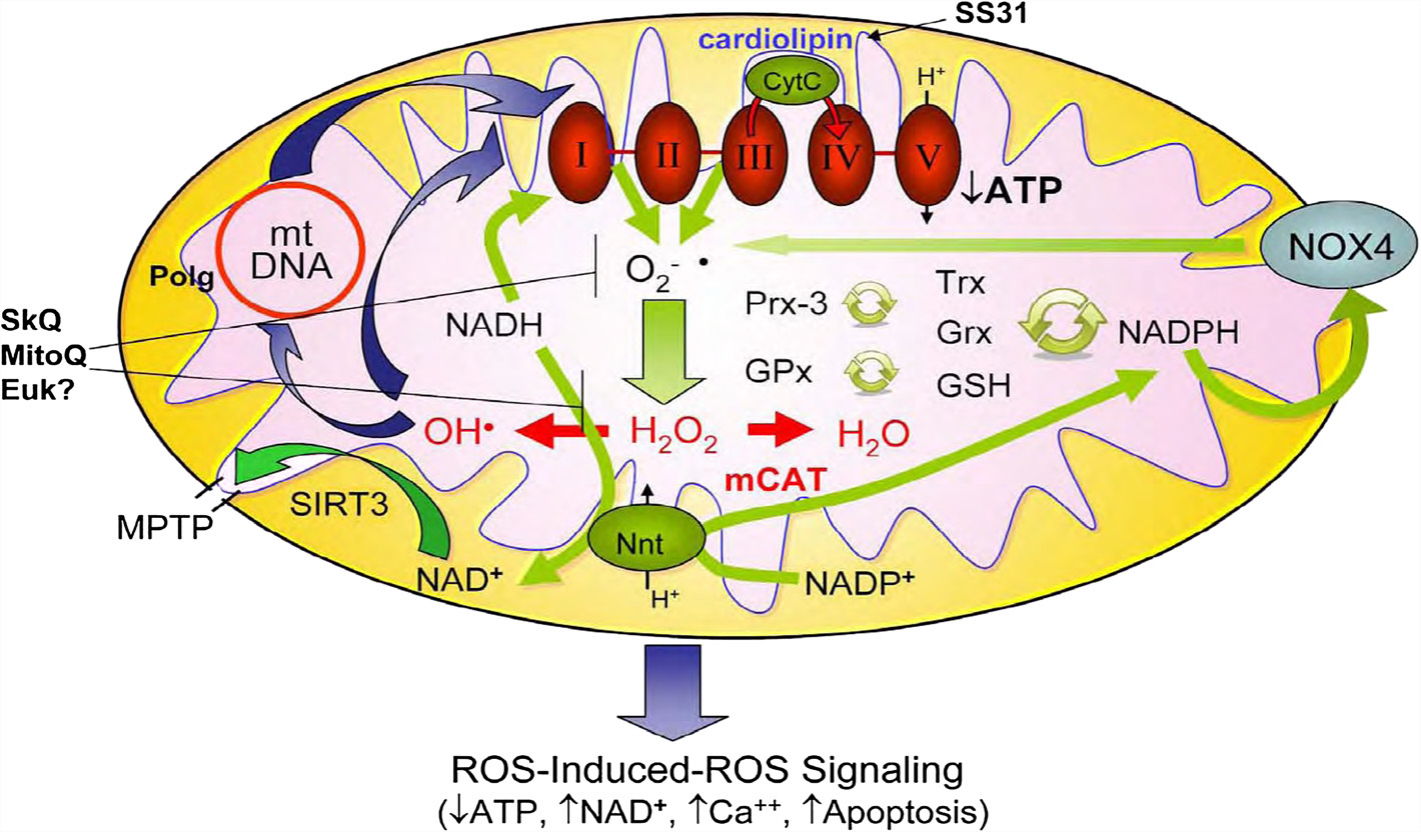
**Interdependences of mtROS, nicotinamide nucleotides, and SIRT3: ROS-Induced ROS Signaling.** Modified from Dai et al. [93].

The lack of anti-aging effect with antioxidant supplements led Harman to modify his original theory to specify mitochondria as both the primary sources of ROS and the primary targets of ROS damage [16]. One of the features of the mitochondrial free radical theory is the central role that mitochondria play in generation of ROS from the electron transport chain, production of energy (ATP), and the numerous potential feedback loops in regulation of mitochondrial and cellular function, in which redox state and ROS might create ‘vicious cycles’ (Figure 2). These include mutations or deletions in mtDNA, which can result in damaged proteins, including important components of the electron transport chain that are encoded by mtDNA, as well as balances in mitochondrial redox state, including glutathione (GSH/GSSG) and nicotinamide dinucleotides. Even these are intertwined, as NADPH is used by glutathione reductase to regenerate glutathione (GSH) from oxidized glutathione (GSSG) (Figure 2). NADPH is also in equilibrium with NADH within mitochondrial through the activity of nicotinamide nucleotide transferase (NNT, also called mitochondrial NAD(P) transhydrogenase). The redox balance of NAD/NADH is the key regulator of the sirtuin histone deacetylases, including mitochondrial SIRT3. The latter has been shown to play a key role in the acetylation state of cyclophilin D, which in turn plays an important role in control of the mitochondrial permeability transition pore (mPTP) and apoptosis (Figure 2).

The revised mitochondrial free radical theory suggests that failures of antioxidants to extend murine lifespan or failure of antioxidant supplements in clinical trials might be explained by poor distribution of antioxidants to mitochondria, and proposes that antioxidants targeted to mitochondria might be beneficial for lifespan extension. Several lines of evidence have supported the mitochondrial theory of aging. One of the most direct experimental evidence for the role of mitochondrial ROS in longevity was shown in mice overexpressing catalase targeted to mitochondria (mCAT), which resulted in a significant median and maximal lifespan extension in two independent lines of C57Bl6 mice [17]. Interestingly, similar overexpression of catalase targeted to peroxisome (pCAT), its normal location within the cell, or nuclear localization (nCAT) had modest and non-significant effects on murine lifespan. This indicates that mitochondrial localization of the catalase is key to lifespan extension in this model [17]. Consistently, mitochondria-targeted antioxidant SkQ1 has been shown to prolong the lifespan of inbred male mice in specific pathogen free (SPF) condition and outbred mice and dwarf hamster in conventional or outdoor cages [18].

Additional evidence for the involvement of mitochondrial ROS in aging comes from observations of mice with a targeted mutation of the p66^Shc^ gene. These mice display reduced ROS generation and increased resistance to ROS-mediated apoptosis and thereby have a prolonged lifespan [19]. Further studies have shown that P66^Shc^ is a mitochondrial redox enzyme located in the mitochondrial intermembrane space, which produces H_2_O_2_ from electron leakage during oxidative phosphorylation [20]. Phosphorylated p66^Shc^ was later shown to accumulate within mitochondria to activate mitochondrial Ca^2+^ response, and subsequently induce apoptosis [21]. Further evidence supporting the role of mitochondria in aging was demonstrated using mice with homozygous mutation in exonuclease domain of the mitochondrial polymerase gamma (Polga^*D257A/D257A*^ , abbreviated Polg^*m/m*^). These mice were susceptible to accumulation of mtDNA point mutations and deletions with age [22,23]. They have shortened lifespan (maximal lifespan approximately 15 months) and display many phenotypes of ‘accelerated aging’, including kyphosis, graying and loss of hair, anemia, osteoporosis, sarcopenia (loss of muscle mass), and presbycusis (age-related hearing loss) [22]. Interestingly, the ‘premature’ aging-like phenotype in these mice showed good correlation with the accumulation of mtDNA deletions but not with the burden of mtDNA point mutations [24]. Moreover, the accumulation of mtDNA damage has been shown to increase apoptosis [23] and age-dependent cardiomyopathy and oxidative damage in the Polg^m/m^ mouse heart was attenuated by mCAT [25].

In spite of these ‘attractive’ aspects of the mitochondrial free radical theory of aging, there remain many unsolved questions. Damage to mitochondrial DNA has been shown to increase with age, even more so than nuclear DNA [26]; however, it is necessary to distinguish point mutations from deletions; for example, it has been argued that the former do not [27] whereas the latter do correlate with lifespan of mice [24] This may be related to the fact that mitochondria have multiple copies of DNA, providing protection from heteroplasmic mutations. Recent improvements in DNA sequencing methods have also revealed the surprising result that age-related increases in mtDNA point mutations in human brains are primarily DNA transitions, whereas oxidative damage is expected to produce an excess of G ➔ T transversions [28]. Deletions, however, appear to accumulate and expand in the population of mtDNA during aging and some pathologies and it appears likely that mitochondrial respiratory failure only occurs with high loads of mtDNA deletion, such as has been observed in muscle fibers, intestinal crypts, and substantia nigra neurons (see review [29]). Finally, there is an increasing awareness that low levels of mitochondrial ROS may be ‘hormetic’ by inducing endogenous antioxidant defenses to prevent oxidative stress induced in pathological states and that mitochondrial ROS may be an important mediator of cell signaling (see section below).

The mitochondrial free radical theory has led to a focus on development and refinement of drugs to specifically target ROS specifically in the mitochondria of cells. The most common approach is based on delivery of known redox agents to the mitochondrial matrix by conjugation to delocalized cations (such as the triphenylphosphonium ion (TPP+)), including MitoQ and SkQ1 [30,31]. The Szeto-Schiller (SS) peptides represent a different chemical approach to reduce mitochondrial ROS. These aromatic-cationic tetrapeptides are targeted to cardiolipin on the inner mitochondrial membrane, and they have been shown to modulate electron flux in the electron transport chain and increases ATP production, while reducing electron leak and inhibiting excessive ROS production [32]. These mitochondria-targeted antioxidants are discussed in greater detail later (see section Mitochondrial protective strategies as potential therapeutics for aging-related diseases).

### Mitochondrial signaling and the theory of mitohormesis

Apart from generating detrimental oxidative damage, ROS have numerous crucial biological roles in signaling and stress response (reviewed in [33-35]). Emerging evidence suggests that oxidative stress might promote longevity and metabolic health through the concept of mitochondrial hormesis (mitohormesis). The mitohormesis theory hypothesizes that low levels of oxidative stress induced by either caloric restriction, exercise [36], or other stimuli may trigger adaptive responses that improve overall stress resistance, probably through increased endogenous antioxidant defense, which may eventually reduce chronic oxidative damage [37] and subsequently achieve lifespan extension. This concept is supported by a study in *C. elegans* demonstrating that inhibition of respiration increases mitochondrial ROS production and significantly increases lifespan via mitochondrial ROS mediated activation of HIF-1 [38]. Low dose of oxidative stress induced by dietary restriction, especially glucose restriction, has been shown to preferentially induce mitochondrial metabolism and extend lifespan in various model organisms, including *Drosophila melanogaster*[39] and *Caenorabditis elegans*[40]. For instance, glucose restriction in *C. elegans* extends lifespan by inducing mitochondrial respiration and increasing oxidative stress, and this AMPK-dependent lifespan extension is abolished by pre-treatment of antioxidant N-acetyl cysteine, suggesting that oxidative stress is required for lifespan extension of dietary restriction [40]. Although the evidence of hormesis in lifespan regulation in mammalian models is still lacking, considerations should be taken when developing antioxidant therapy.

The theory of mitohormesis could have important translational implications as an ideal antioxidant therapy might be one that prevents oxidative damage induced under pathological conditions without interfering with ROS needed for hormesis and cellular signaling. We speculate that the targeted expression of catalase in mitochondria (mCAT) might be such an example, as there are beneficial effects of mCAT in aging and several disease models with negligible adverse effects (Table 1). Key to this may be that the Km of the catalytic activity of catalase is >10 mM, so that this enzyme is less likely to be effective at the lower intracellular H_2_O_2_ concentrations that may be involved in signaling or hormesis [41,42].

**Table 1:** Mitochondrial targeted genetic and pharmacological manipulations on aging and healthspan

	**Animal models**	**Description**	**Aging phenotypes**	**Healthspan phenotypes**
**Genotypes**	mCAT	Overexpression of catalase targeted to mitochondria	18% extension of lifespan [17]. Attenuated cardiac aging [43], aging-related sarcopenia [17], presbyacusis [44], and cancer incidence [45].	Protect against cardiac hypertophy and heart failure [46]
				Reduce Aβ toxicity and oxidative injury, and extends the lifespan of Aβ PP overexpressing mice [47]
				Protective against mitochondrial ROS production and subsequent dopaminergic neuron degeneration in MPTP-induced Parkinson’s disease model [48]
				Attenuate lipid-induced insulin resistance in skeletal muscle [49]
	Polg^m/m^	Homozygous mutation of mitochondrial polymerase gamma D257A	‘Accelerated aging’: sarcopenia, graying and alopecia, kyphosis, presbyacusis, anemia [22,23], age-dependent cardiomyopathy [25]	Aggravate heart failure in response to Angiotensin II [46]
	p66^shc^	Targeted mutation of the p66^Shc^ gene	Extension of lifespan. Reduction of ROS and apoptosis [19]	Attenuate Angiotensin II induced LV hypertrophy and cardiomyocytes apoptosis; reduce oxidative damage in cardiac progenitor cells, cardiomyocytes and endothelial cells in diabetes [19,21,50,51]
	SIRT3^-/-^	SIRT3-deficient mice	Accelerated cardiac aging, age-dependent increase in mitochondrial swelling due to increased mPTP opening [52]	Early-age onset of hypertrophy associated with fibrosis
			Abolish CR effect in reduction of oxidative damage, protection of cochlear neurons and prevention of presbycusis [53]	Increased mortality after transverse aortic constriction [52]
**Pharmacological treatments**	SS-31	Mitochondrial protective tetrapeptide	Reverse age-related muscle weakness and muscle energy deficits [54]	Attenuation of Angiotensin II induced cardiac hypertrophy and Gαq overexpression induced heart failure [55]
				Ameliorate cardiac dysfunction after tranverse aortic constriction [56]
				Improve systolic function ischemic HF model [57,58]
				Attenuate cardiac I/R injury [59,60]
				Protect against renal I/R injury [61]
				Prevent high fat diet induced insulin resistance in skeletal muscle [62]
				Attenuation of diabetic retinopathy [63]
				Protective against ALS in SOD1 mutant mice [64] and Parkinson’s diseases in MPTP model [65]
	MitoQ	Ubiquinone (antioxidant) conjugated with TPP+		Reduction of blood pressure and cardiac hypertrophy in spontaneous hypertensive rats [66]
	SkQ	Plastoquinone conjugated with TPP+	Prolonged lifespan. Attenuation of age-related decline in immunity. Protective against baldness and lordokyphosis in aged mice [18,67]	Attenuate heart arrhythmia, I/R injury, myocardial infarction, and kidney ischemia [68]
				Delayed tumor development in p53-deficient mice [30]
				Protect against cataract and retinopathy in OXYS rats [69]

### Mitochondrial oxidative stress in healthspan

#### Cardiac aging

Increasing evidence suggests that abnormal mitochondrial ROS (mtROS) production and detoxification contributes to mitochondrial dysfunction and cardiomyopathy in old age (reviewed in [35,70,71]). An age-dependent reduction in cardiac mitochondrial oxidative phosphorylation function is related to the decline in mitochondrial state 3 respiration (maximal stimulated respiration) due to diminished activity of electron transport complexes I and IV (both have subunits encoded by mtDNA), while complexes II, III, and V are relatively unaffected (see review [72]). Impaired electron transport chain function is directly related to elevated electron leakage and generation of mtROS. Since the heart has a high metabolic demand and is rich in mitochondria, it produces ROS within mitochondria as a byproduct of oxidative phosphorylation and is, therefore, especially susceptible to oxidative damage. It has been shown that mitochondrial production of ROS significantly increases in the heart with advanced age [73].

**Figure 3:**
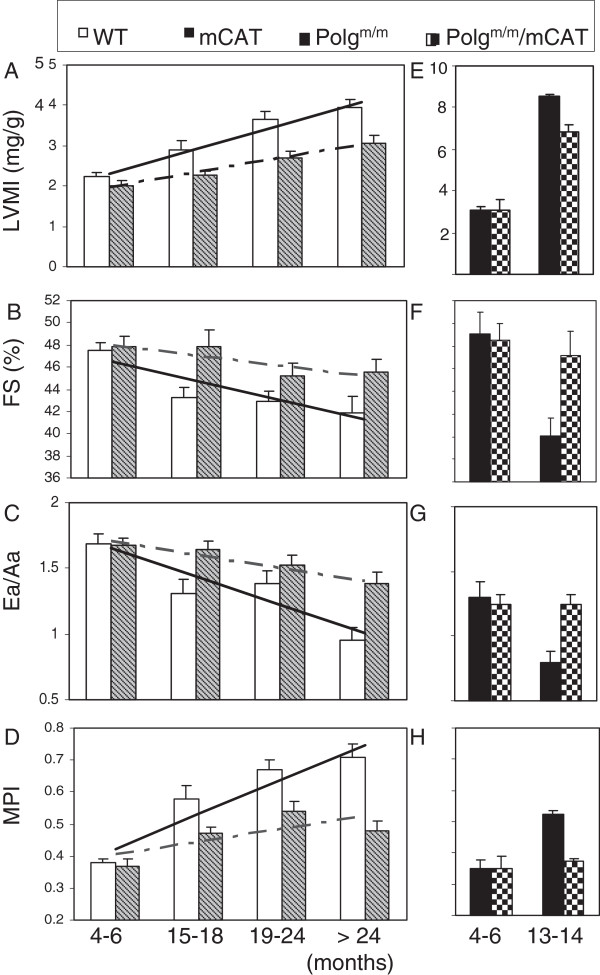
**Echocardiography of cardiac aging in wild-type (WT) and mCAT mice (A-D) and Polg**^**m/m **^**mice in the presence or absence of mCAT (E-H). (A, E)** Left ventricular mass index (LVMI), **(B, F) **% FS (fractional shortening), **(C, G)** Ea/Aa by tissue Doppler imaging (diastolic function), **(D, H)** the myocardial performance index (MPI). The increased linear trends across ages in WT mice were significant for all parameters (*P* <0.05 for all, left panels). The beneficial effect of mCAT *versus* WT was analyzed by the interaction between genotype and the linear age trend, and was significant in all cases (*P* <0.01 for all except fractional shortening, *P* = 0.03). *P <0.05 *versus* Polg^m/m^ at age 4 to 6 months, #*P* <0.05 *versus* Polg^m/m^ at age 13 to 14 months (right panels). LVMI, Left ventricular mass index; mCAT, catalase targeted to mitochondria. Modified from Dai et al. [25,43].

The Framingham Heart Study and the Baltimore Longitudinal Study on Aging (BLSA) demonstrate that aging is associated with increased prevalence of left ventricular hypertrophy and decline in diastolic function (measured by the ratio of early to late ventricular filling (E/A) by Doppler echocardiography) in otherwise healthy individuals. Left ventricular (LV) wall thickness increases and maximal exercise capacity decreases with age in both sexes, indicative of LV hypertrophy, while systolic function is relatively preserved at rest (reviewed in [74,75]). Cardiac aging in murine models closely recapitulates those seen in humans [76], including cardiac hypertrophy (Figure 3A), a modest decline in systolic function (%FS, Figure 3B), a significant decline in diastolic function measured by Ea/Aa (Figure 3C), and worsening of the myocardial performance index (that is, an increased fraction of systole was spent during ineffective isovolumic contraction and relaxation, Figure 3D) [43]. The proportion of mice with diastolic dysfunction and left atrial dilatation also significantly increased with age [43].

**Figure 4:**
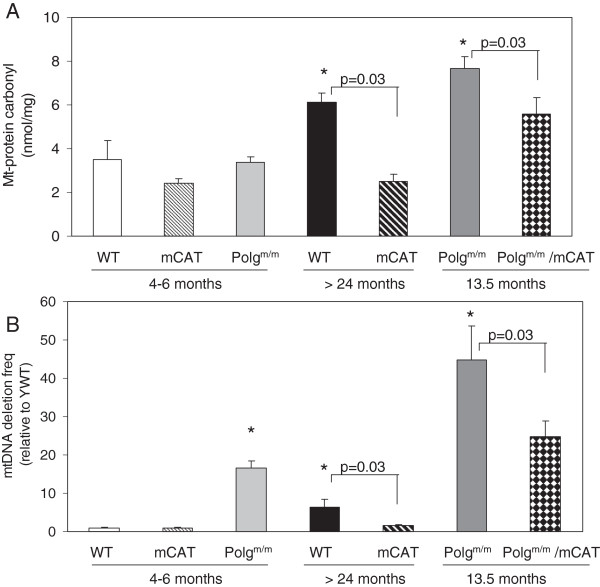
**Mitochondrial oxidative damage and mtDNA deletions in cardiac aging. (A)**. Mitochondrial protein carbonyl (nmol/mg) significantly increased in old wild-type (OWT, >24 months) and even more in middle-aged Polg (13.5 months) mouse hearts when compared with young WT mouse hearts. mCAT significantly reduced the age-dependent mitochondrial protein carbonylation. **(B)** Mitochondrial DNA deletion frequency significantly increased in OWT (>24 months) and young Polg (4 months) when compared with young WT, and this is dramatically increased in middle-aged Polg (13.5 months). mCAT overexpression significantly reduced the deletion frequency for both. *P <0.05 compared with YWT. Modified from Dai et al. [25,43].

Data from our laboratory demonstrated that mCAT greatly attenuated many of these cardiac aging phenotypes (Figure 3A-D). The preserved cardiac aging phenotypes in mCAT mice were accompanied by reductions of age-dependent increases in mitochondrial protein carbonyls (Figure 4A) and mtDNA deletions (Figure 4B), suggesting prevention of mitochondrial oxidative damage as a mechanism of the cardiac aging protection. The success of mCAT protection in cardiac aging and the inability to confer similar protection by overexpression of peroxisomal catalase or the non-targeted antioxidant N-Acetyl Cysteine [55] underscores the importance of mitochondrial specificity in antioxidant intervention. Given the complexity of the systems involved, it is likely that mitochondrial dysfunction and aberrant ROS production may contribute to aging through both direct damage to cellular macromolecules and interference with normal signaling and energetics. There is an age-dependent increase in electron leakage and superoxide production. This makes a positive feedback between complex I inhibition and mitochondrial ROS production, as well as the more classical vicious cycle of mitochondrial DNA mutation and protein damage amplifying ROS (Figure 2). The effect of mitochondrial ROS in signaling and energetics may be a critical factor in cardiac (and other organ system) aging.

As discussed above, mice with homozygous mutation of mitochondrial polymerase gamma (Polg^*m/m*^) have substantial increases in mtDNA mutations and deletions with age [22,23], shortened lifespan and exhibit several progeroid phenotypes, and developed cardiomyopathy in middle age (13 to 14 months) [22,25]. Middle age Polg^*m/m*^ mice display cardiac hypertrophy (Figure 3E) and impaired systolic and diastolic function (Figure 3F-G) to an extent that is more severe than wild-type (WT) mice aged 24 to 30 months. Interestingly, mCAT partially rescues the mitochondrial damage and cardiomyopathy in Polg^*m/m*^ mice (Figure 3E-H), supporting the role of mitochondrial ROS and mtDNA damage as part of a vicious cycle of ROS-induced ROS release (Figure 2) [25]. An interesting study shows that endurance exercise can prevent both skeletal muscle and cardiac progeroid phenotypes in Polg^m/m^ mice [77]. The beneficial effect of exercise is thought to be mediated by the augmented level of mitochondrial biogenesis seen with exercise in these mice, which contributes to the preserved mitochondrial and, subsequently, organ function. Exercise induces ROS, and ROS stimulates upregulation of PGC1α [78], which is the master regulatory molecule in mitochondrial biogenesis and is known to improve endogenous antioxidant systems. The beneficial effect of exercise in this scenario is indeed a good example of mitochondrial hormesis mentioned above.

#### Cardiovascular diseases

Separate from the intrinsic decline in cardiac function during healthy aging mentioned above, old age is associated with an exponential increase in the prevalence of hypertension, stroke, coronary heart disease, and heart failure, especially in people aged over 65 years. Increased oxidative stress has been implicated in the pathogenesis of cardiovascular diseases, including hypertension, atherosclerosis, cardiac hypertrophy due to cardiac aging or pressure overload, cardiac ischemia-reperfusion injury, as well as cardiac failure. As in cardiac aging, a deficiency of mitochondrial energetics has been documented in human and experimental animals with heart failure [79]. Mechanisms may include mitochondrial biogenesis that does not keep up with the increasing demand (see review [80]), mitochondrial uncoupling and decreased substrate availability [81], and increased mitochondrial DNA deletions [46]. Mutations of genes encoding mitochondrial enzymes have been shown to be associated with various forms of idiopathic hypertrophic and dilated cardiomyopathies [82]. Mitochondrial DNA deletions have been found in experimental models of heart failure [83]. Studies on human hearts using ^31^P NMR spectroscopy indicated that the ATP content of failing hearts is generally 20% to 30% lower than that of normal hearts [84]. Furthermore, phosphocreatine, an important short-term reserve energy source that maintains a high phosphorylation potential to cope with acute increases in energy demand (for example, exercise), significantly declined by up to 60% in elderly heart failure patients [85]. The magnitude of this reduction is related to the severity of heart failure [86] and is shown to predict mortality in patients with dilated cardiomyopathy [87].

Hypertension is the most common cause of cardiac hypertrophy, which predisposes to chamber dilatation, heart failure, and sudden cardiac death [88]. Angiotensin II, a key molecule in the Renin-Angiotensin System which regulates hypertension, is well known to cause left ventricular hypertrophy and fibrosis [89]. At the molecular level, Angiotensin II binds to ATR1, a Gαq coupled-receptor, then activates NADPH oxidase through a PKC-dependent manner to produce ROS [90]. ROS from NADPH oxidase, particularly the NOX4 isoform, might increase mitochondrial ROS production, as previously shown in endothelial and vascular smooth muscle cells [91,92]. Mechanisms of ROS amplification in mitochondria might include ROS induced ROS release as well as a ROS-mtDNA damage vicious cycle (Figure 2) (see review [93]).

A study from our laboratory showed that Angiotensin II delivered for 4 weeks by an osmotic minipump induced increased blood pressure, cardiac hypertrophy, cardiac fibrosis, and diastolic dysfunction [55]. This experimental model of cardiac hypertrophy is associated with increased cardiac mitochondrial protein carbonyl content and the frequency of mitochondrial DNA deletions, indicating oxidative damage to mitochondria [46]. The accumulation of mitochondrial oxidative damage activated mitophagy, which in turn increased signaling for mitochondrial biogenesis through activation of peroxisome proliferator-activated receptor gamma coactivator-1 alpha (PGC-1α) and its target genes. Our observation is consistent with the report that PGC-1α is transcriptionally upregulated by ROS [78]. mCAT, but not pCAT, were resistant to cardiac hypertrophy, fibrosis, and diastolic dysfunction induced by Angiotensin II [46]. This strongly supports a central role of mitochondrial ROS in Angiotensin II-induced cardiomyopathy [46]. Additional evidence from other laboratories show that disruption of p66^Shc^ prevents Angiotensin II-induced LV hypertrophy and cardiomyocyte apoptosis as well as reducing oxidative damage in cardiac progenitor cells, cardiomyocytes, and endothelial cells in a diabetic mouse model [50,51,94]. Moreover, mice deficient in mitochondrial deacetylase SIRT3 displayed early age onset of hypertrophy associated with fibrosis, age-dependent increase in mitochondrial swelling due to increased mPTP opening, increased mortality after transverse aortic constriction [52].

As noted above, Polg^*m/m*^ mice have increased mitochondrial DNA mutations and develop heart failure at middle age or at young age when challenged with Angiotensin II, both of which are attenuated by mCAT [43,46]. This suggests that primary damage to mitochondrial DNA contributes directly to the phenotype of systolic heart failure, through increased mt ROS. Therefore, the protective effects of mCAT expression in Ang-induced cardiac hypertrophy and Gαq-induced heart failure provide direct evidence that amplification of ROS within mitochondria is a key mediator in these disease models [46]. Using the transverse-aortic constriction (TAC) mouse model, we further show that TAC-induced heart failure is associated with remodeling of the mitochondrial proteome, including decreased abundance of proteins involved in fatty acid metabolism and increased abundance of proteins in glycolysis, apoptosis, mitochondrial unfolded protein response, and proteolysis. Overexpression of mCAT mitigates the phenotype of heart failure, better preserves proteins involved in fatty acid metabolism, and attenuates the increases in apoptotic and proteolytic enzymes [95]. Thus, breaking the ROS vicious cycle within mitochondria by mCAT is effective in attenuating both cardiac hypertrophy and failure (Figure 2). In a highly parallel manner we also demonstrated that the mitochondrial protective peptide SS31 attenuates cardiac hypertrophy and diastolic dysfunction induced by chronic Angiotensin II, and the heart failure phenotypes induced by overexpression of Gαq or transverse aortic constriction (See section Mitochondrial protective strategies as potential therapeutics for aging-related diseases). Furthermore, SS-31 has also been shown to prevent hypoxia-reoxygenation induced apoptosis in renal tubular epithelial cell by downregulation of p66^Shc^[96].

Ischemic-reperfusion (I/R) injury often occurs during acute myocardial infarction, either due to spontaneous recanalization of the occluded artery or as a result of a reperfusion therapy. ROS are well known to be primary mediators in IR injury. ROS begin to accumulate during ischemia [97], causing mitochondrial respiratory complex dysfunction, which leads to a burst of ROS after reperfusion. Furthermore, post-ischemic reperfusion is associated with ROS accumulation, acidic pH, and a rise in [${\mathrm{Ca}}_{i}^{\mathrm{2+}}$], conditions which have been shown to open the mPTP, which in turn triggers more mitochondrial ROS generation. This is one of the mechanism involved in mitochondrial ROS-induced ROS release [98] (Figure 2).

The aged myocardium has less tolerance to ischemia and hemodynamic stress than the young myocardium [99]. Aged cardiomyocytes have a lower threshold for ROS induced ROS release and increased susceptibility to mPTP opening [100]. Ischemic preconditioning is also impaired in the aged myocardium (reviewed by [100]). This loss of endogenous protective mechanisms of ischemic preconditioning in the aged heart might be due to a decrease in mitochondrial heat shock protein-70 [101], reduced nitric oxide bioavailability [102], damaged mitochondria that are vulnerable to stress, and diminished PKC translocation into mitochondria, all of which are required for the protective effect of ischemic preconditioning [103,104]. Cardiac aging and various models of cardiomyopathy in the context of mitochondrial ROS are summarized in Table 1.

#### Skeletal muscle aging

Sarcopenia is the loss of skeletal muscle mass and function with age. Sarcopenia is an important public health concern due to its role in exercise intolerance, increased morbidity, and loss of independence in the elderly [105-108]. This loss of independence is due to an inability to perform activities of daily living that require sustained muscle power, such as walking, dressing, and showering as well as an increased risk of falling [109]. The resulting increased rates of nursing home placement and hospitalization make the loss of skeletal muscle function with age a growing public health crisis in terms of both quality of life and economic costs to society. Janssen et al. [110] estimated these costs at $18 billion dollars in 2001 and predicted that a 10% reduction in sarcopenia prevalence would lead to a savings of $1.4 billion in healthcare costs (adjusted to 2010 dollars) [110].

Skeletal muscle, like heart, relies on mitochondria to meet the majority of the ATP demands for sustained muscle contraction. Mitochondrial function in skeletal muscle is very dynamic where the metabolic rate can vary by at least an order of magnitude during rest to work transitions, as well as varying with nutritional state. One consequence of this variation in mitochondrial function is that periods of increased mitochondrial ROS production are a normal part of the physiology of skeletal muscle [62,111]. Skeletal muscles also produce significant ROS from non-mitochondrial sources, primarily sarcolemmal NAD(P)H oxidases [112], that can also contribute to increased cellular and mitochondrial oxidative stress. These transient increases in oxidative stress modify muscle function and may play an important role in the beneficial adaptations to exercise training [36,113]. However, mitochondria in aged skeletal muscle have an increased capacity to produce H_2_O_2_ when measured under *ex vivo* conditions [54]. This increased mitochondrial oxidative stress can control mitochondrial function both *in vivo*[114,115] and *ex vivo*[116,117]. Inducing a mild oxidative stress in adult mice for 24 h using low doses of paraquat recapitulates the reduced mitochondrial coupling (P/O) and depression of skeletal muscle metabolism [114,115] observed *in vivo* in aged skeletal muscle in both mice [118] and humans [119]. This same paraquat treatment in old mice led to decreases in maximal mitochondrial ATP production (ATPmax), in addition to further decreases in P/O and resting metabolism [115]. This increased sensitivity is consistent with a decline in the ability of the aged skeletal muscle to buffer transient increases in oxidative stress.

**Figure 5:**
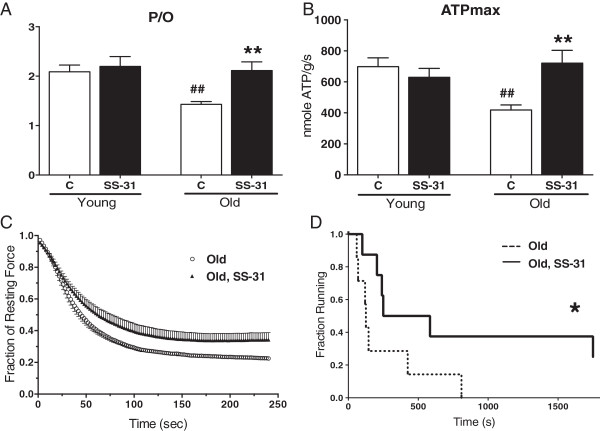
**Mitochondrial targeted SS-31 improves skeletal muscle function.***In vivo* mitochondrial coupling ratio (P/O) **(A)** and maximum mitochondrial ATP production **(B)** in the hindlimb muscles of aged mice were both increased 1 h after treatment with SS-31. *In situ* fatigue resistance in the aged mice was also increased 1 h after SS-31 treatment **(C)**. Eight days of daily treatment with SS-31 led to increased endurance capacity in the aged mice **(D)** as well. Means ± SEM. n = 5-7 per group. **P <0.01 relative to age-matched control. ##*P* <0.01 relative to young control. Young - 5 months old; Old - 27 months old. Modified from Siegel et al. [54].

Further support for a contribution of mitochondrial oxidative stress in age-related skeletal muscle dysfunction comes from experiments using the mitochondrial targeted peptide SS-31. SS-31 accumulates in the mitochondria by associating with the inner mitochondrial membrane [61] and reduces mitochondrial H_2_O_2_ production [54,62]. One hour after treatment with SS-31 age-related declines in mitochondrial P/O, ATPmax, and skeletal muscle metabolism were reversed (Figure 5) [54] and the skeletal muscle glutathione redox state was more reduced [120]. These metabolic changes were associated with improved fatigue resistance of the tibialis anterior muscle *in situ* and increased endurance capacity in the aged mice.

Genetic manipulation of mitochondrial antioxidants also supports a role for mitochondrial oxidative stress in controlling skeletal muscle function and metabolism. Deficiency of MnSOD (mitochondrial specific isoform of superoxide dismutase) in type IIB muscle fibers leads to an increase in mitochondrial oxidative stress and dysfunction in fast-twitch mouse muscles. Mitochondria from fast-twitch muscles in these mice had significantly reduced aconitase and succinate dehydrogenase (complex II of the electron transport chain) activities and increased capacity for superoxide production resulting in elevated F_2_-isoprostanes [121]. Both fatigue resistance in isolated muscles and whole body endurance performance were also decreased in the MnSOD deficient mice. Interestingly, MnSOD deficiency did not lead to an increase in muscle atrophy or change in maximal force production in aged mice. Conversely, mCAT preserved mitochondrial function and insulin sensitivity in skeletal muscle of aged mice [49], while vector delivery of mCAT into embryos led to an increase in exercise performance in 3-month-old mice [122]. However, the ectopic expression of mCAT had no effect on the contractility and fatigue resistance in isolated extensor digitorum longus muscle. The lack of effect in the isolated muscle may indicate that the increased exercise tolerance in this study was due to improved cardiac function as described above. Alternatively, the mosaic expression of mCAT in the skeletal muscles may have limited its effect on *ex vivo* skeletal muscle performance.

The current evidence strongly supports an important role of mitochondrial oxidative stress in the decline in skeletal muscle function with age, while its role in skeletal muscle atrophy with age is still controversial. The strongest evidence in support of a role for oxidative stress in age-related muscle atrophy comes from mice lacking CuZnSOD (SOD1). CuZnSOD is found in both the cytosol and the inner membrane space of the mitochondria. The absence of CuZnSOD leads to an accumulation of peroxinitrite and oxidative damage [123], increased mitochondrial ROS production, increased sensitivity to apoptotic loss of myonuclei, and mitochondrial dysfunction associated with a premature loss of skeletal muscle mass in aging mice [12]. Muscle fibers from CuZnSOD^-/-^ knockout mice accumulated mitochondria around the neuromuscular junction, showed a loss of motor units and disruption of neuromuscular junctions. However, muscle specific knockout of CuZnSOD did not result in muscle atrophy, increased oxidative stress, nor mitochondrial dysfunction [124], but did have a loss of specific force throughout life. The lack of atrophy in the skeletal muscle specific deficiencies in CuZnSOD and MnSOD knockouts led these authors to suggest that increased oxidative stress in the myofibers are not primary cause of muscle atrophy with age. Instead they suggest that increased oxidative stress in the motorneurons leading to denervation may be the primary driving force behind loss of muscle mass. This conclusion is supported by the observation that direct stimulation of skeletal muscles in the CuZnSOD^-/-^ mice leads to significant increases in force production over that achieved by nerve stimulation [124]. This result suggests that force is limited not by the muscle itself, but by the ability of the motorneuron to maximally stimulate the available myofibers. Thus, the data from the CuZnSOD^-/-^ mice suggest an important role of increased oxidative stress in age-related muscle atrophy, although it remains unclear whether oxidative stress originating in the myofibers or the motorneurons are the primary drivers of this process. This conclusion is supported by recent work demonstrating that treatment with SS-31 during hind limb unloading ameliorated muscle atrophy and mitochondrial dysfunction [125].

There are multiple ways in which an increase in oxidative stress can affect skeletal mitochondrial and contractile function. Work to date has primarily focused on the accumulation of oxidative damage to proteins, lipids, and DNA. Most studies find a clear accumulation of oxidatively damaged macromolecules with age in skeletal muscle and most other tissues. However, oxidation of the mitochondrial and cellular redox environment can also exert control over cellular function through post-translational modification of proteins (Figure 1). Glutathionylation is a key redox dependent post-translational modification in the mitochondria. Increased oxidative stress has been found to lead to increased glutathionylation and inhibition of activity of electron transport chain proteins, F_1_F_0_ ATPase [126] and complex I [127], and of TCA cycle proteins, succinyl-CoA-transferase [126] and α-ketoglutarate dehydrogenase [128]. In addition recent evidence indicates that glutathionylation of UCP3 regulates proton leak under conditions of acute oxidative stress in skeletal muscle [129] resulting in increased proton leak and reduced P/O.

Redox modification of proteins can also affect contractile function. The skeletal muscle ryanodine receptor 1 (RyR1) in aged rats is oxidized by cysteine nitrosylation [130]. This leads to a loss of RyR1-calstabin1 interaction, destabilization of the channel and increased calcium leak from the SR, which causes a loss of specific force and reduced exercise tolerance with age. Stabilizing the channel and preventing calcium leak rescued force production and exercise tolerance. As pointed out by the authors, leaky RyR would lead to elevated cytosolic calcium and increased calcium loading by the mitochondria, and an elevation of mitochondrial ROS production. This could then lead to a feed-forward mechanism further exacerbating skeletal muscle dysfunction. Thus, oxidation of the cellular redox status in aged muscle [131] can contribute to energetic and contractile deficits through both reversible and post-translational modification of proteins.

#### Neurodegenerative disease

Old age is associated with progressive decline in the functional performance of the nervous system. This intrinsic nervous system aging includes slowed reaction times, degeneration of sensory and motor function, and a decline in cognitive performance. In addition to intrinsic nervous system aging, several neurodegenerative diseases demonstrate strong age-related onset including the highly prevalent Alzheimer’s disease (AD) and Parkinson’s disease (PD), among others.

#### Age-related sensorineural hearing loss

Age-related sensorineural hearing loss or presbycusis is gradual loss of hearing with aging. The prevalence in the elderly is estimated to be 30% to 35% of people aged 65 to 75 years and 40% to 50% of people aged older than 75 years [132]. The sensorineural hearing loss is usually more severe for high pitched sound, which eventually leads to difficulty in understanding speech. The pathology is characterized by age-dependent loss of sensory hair cells, spiral ganglion neurons, and stria vascularis cells in the inner ear cochlea. Someya et al. [44] reported that mice with the deletion of the mitochondrial pro-apoptotic gene Bak attenuated age-related apoptotic cell deaths and hence prevented presbycusis. While oxidative stress induced Bak expression in primary cochlear cells, mCAT suppressed Bak expression, reduced cell death and subsequently prevented presbycusis. These findings suggest a central role of mitochondrial ROS induced apoptotic pathway in presbycusis [44]. They further demonstrate that caloric restriction prevents presbycusis via reduction of oxidative damage by mitochondrial deacetylase SIRT3. In response to CR, SIRT3 directly deacetylates and activates mitochondrial isocitrate dehydrogenase 2, leading to increased NADPH levels and an increased ratio of reduced-to-oxidized glutathione in mitochondria and thereby enhancing the mitochondrial glutathione antioxidant defense system [53].

#### Alzheimer’s disease

AD is the most prevalent neurodegenerative disease, affecting approximately 5 million Americans. The clinical presentation of AD is primarily memory impairment and dementia. The early memory deficit in AD is often described as ‘recent memory impairment’ (for example, inability to recall a couple of words after a few minutes of distraction) [133]. Deficits in other cognitive functions may appear later after the development of memory impairment.

There are two principle pathologic lesions in AD: neurofibrillary tangle (NFT) and the amyloid plaque [134]. The NFTs consist of abnormal accumulations of abnormally phosphorylated tau within the cytoplasm of certain neurons. The amyloid plaques contain β-amyloid peptide (Aβ), which arises through proteolytic processing of amyloid precursor protein (APP) by β-secretase and γ-secretase (presenilin1/2). Each of these lesions has a characteristic distribution. The hierarchical pattern of NFTs among brain regions is so consistent that a staging scheme based on the topography of these lesions has been widely used [135]. The majority of AD cases are sporadic and occur very late in life, however, less than 1% of AD is familial AD cases, which have an early onset and are inherited in an autosomal dominant manner. Genes implicated in the early onset AD include β-amyloid precursor protein (APP), presenilin 1, and presenilin 2 [136]. Mutation of the APP gene affects the cleavage of APP by β-secretase or γ-secretase to generate various forms of Aβ. The Aβ peptides have a tendency to form oligomer aggregates and become toxic, especially the long form, Aβ_1-42_. Presenilins are integral membrane proteins that function as the proteolytic components of γ-secretase. Mutations of presenilins result in increased production of Aβ_1-42_.

Several lines of evidence have shown the central roles of mitochondria in AD (see reviews [137]). Both APP and presenilin have been isolated in mitochondrial fraction [138,139]. Moreover, Aβ is imported into the mitochondrial cristae through translocase of outer mitochondrial membrane complex (TOMM) [140]. Increased mitochondrial oxidative stress and damage to mitochondrial structural components and enzyme complexes are well documented in early AD [141-144]. One of the mechanisms involves Aβ, which inhibits mitochondrial function by inhibition of electron transport chain activity, especially complex III and IV, that further leads to increased ROS production, decreased ATP production, and facilitation of cytochrome c release [143,145,146]. Additional insults to mitochondria include altered Ca^2+^ homeostasis [147], increased mitochondrial DNA mutations, and deletions [141]. Furthermore, alterations of mitochondrial dynamics have been implicated in AD [148-150]. It has been shown that S-nitrosylation of Drp1 (a mitochondrial fission protein) mediates β-Amyloid-related mitochondrial fission and neuronal injury [148]. Increased production of Aβ interacts with Drp1, which is a critical factor in mitochondrial fragmentation, abnormal mitochondrial dynamics, and synaptic damage [150].

More direct evidence of the role of mitochondrial oxidative stress in AD is demonstrated by studies using mCAT mice or mitochondrial targeted antioxidants. Mao et al. [47] showed that mCAT decreases amyloid-beta (Aβ) toxicity and oxidative injury, and extends the lifespan of Aβ precursor protein (PP) overexpressing mice. This data provides direct evidence that mitochondrial oxidative stress plays a primary role in AD pathology, and supports the possibility that mitochondria-targeted antioxidants might be an effective therapeutic approach to treat patients with AD.

AD is associated with neuronal cell death, loss of synapses, as well as mitochondrial abnormalities. Incubation of N_2_a cells with Aβ led to reduced neurite outgrowth, lower cell viability, mitochondrial dysfunction, and fragmentation and loss of ATP, all of which were partially protected by simultaneous incubation with either SS-31 or MitoQ [125]. Primary neurons from the AβPP mouse model showed increased H_2_O_2_ production, reduced cytochrome oxidase activity, and decreased ATP levels [151]. There was also decreased anterograde mitochondrial movement, increased mitochondrial fission, and decreased fusion. Treatment with SS-31 restored mitochondrial transport and synaptic viability, and decreased the percentage of defective mitochondria [152].

#### Parkinson’s disease

PD, the second most common neurodegenerative disorder, is characterized by defects in motor functions, manifested as resting tremor, bradykinesia, rigidity, and postural instability. The hallmark pathology of PD is a gradual loss of pigmented dopaminergic neurons in the substantia nigra pars compacta, and accumulation of Lewy bodies in catecholaminergic neurons of the brainstem in the substantia nigra and locus ceruleus. Lewy bodies are abnormal aggregates of proteins composed predominantly of α-synuclein and ubiquitin.

There is substantial support for the central role of mitochondria in the pathogenesis of PD. A few genetic loci have been mapped in rare familial PD cases, and are sequentially named PARK1 to PARK11 (review in [153]). Several genes associated with familial PD have been identified in these loci, and the majority of them are related to mitochondria. PARK1 gene encodes α-synuclein, which has been implicated in the maintenance of mitochondrial membranes [154]. Increased amount of α-synuclein binding to mitochondria inhibits mitochondrial fusion and thereby triggers PD pathology, which can be rescued by PINK1, Parkin, and DJ-1 [154]. PARK8 encodes the leucine-rich repeat kinase 2 (LRRK2) and its mutations have been associated with mitochondrial oxidative phosphorylation dysfunction [155]. LRRK2 regulates mitochondrial dynamics by a direct interaction with DLP1, a mitochondrial fission protein [156]. PARK7 encodes DJ-1, the mutation of which is associated with complex I defects, increased mitochondrial ROS, reduced mitochondrial membrane potential, altered mitochondrial morphology, and dynamics [157-159]. PARK2 and PARK6 encode parkin and PTEN-induced kinase 1 (PINK1), which is involved in mitochondrial dynamics (fusion/fission) and turnover by mitophagy [160-162].

Several rodent models have been used to recapitulate pathology and pathophysiology of PD, including mice with genetic manipulation of many of the genes mentioned above and rodents treated with environmental toxins (see review [163]). Of note, the majority of the environmental toxins that recapitulate PD are mitochondrial complex I inhibitors, such as 1-methyl-4-phenyl-1,2,5,6-tetrahydropyridine (MPTP), paraquat, or rotenone. Inhibition of complex I is associated with impaired mitochondrial respiration and leads to increased mitochondrial ROS production, increased oxidative damage to proteins, lipids, and DNA, which may further activate mitochondrial-dependent apoptotic pathways and cause dopaminergic neuronal cell death.

Direct evidence of the role of mitochondrial ROS in PD was shown by the protective effect of mCAT mouse brains against MPTP induced mitochondrial ROS production and subsequent dopaminergic neuron degeneration in substantia nigra pars compacta [48]. In contrast, harlequin mice with partial deficiency of apoptosis inducing factor, which is required for maintenance of complex I oxidative phosphorylation activity, are more susceptible to MPTP-induced dopaminergic neuronal cell death. The increased sensitivity of harlequin mice to MPTP is reversed by the antioxidant tempol (superoxide dismutase-mimetic) [48]. SS-31 was also shown to dose-dependently protect dopaminergic neurons and preserve striatal dopamine levels in mice treated with MPTP, with complete protection observed at 5 mg/kg [65]. Furthermore, SS-31 prevented MPP^+^-induced inhibition of oxygen consumption, ATP production, and mitochondrial swelling in isolated mitochondria. MitoQ was also reported to be protective against MPTP toxicity [164].

#### Insulin resistance, diabetes, and its complication

Growing evidence has implicated the involvement of oxidative stress in insulin resistance and the pathogenesis of diabetes. Hyperglycemia is associated with increased ROS production from glucose autoxidation, advanced glycosylation end-products (AGEs) formation, polyol pathway, and ROS-producing enzymes including NADPH oxidase [165]. Nishikawa et al. showed that, under hyperglycemia, increased glycolysis generates excess pyruvate, which overloads the mitochondria and leads to superoxide generation from the electron transport chain [166]. This mitochondrial superoxide production triggers the feed-forward cycle of mitochondrial ROS production in diabetes.

The involvement of mitochondrial oxidative stress in muscle insulin resistance has been demonstrated by the protection of mCAT mice in age-related reductions in mitochondrial function and lipid-induced insulin resistance in skeletal muscle [49]. This protection is associated with reduced mitochondrial oxidative damage and preserved mitochondrial respiration in muscle of old mCAT mice. In another study, Anderson and colleagues showed both mCAT mice and WT mice treated with SS-31 have reduced high-fat diet-induced mitochondrial H_2_O_2_ emission and showed preserved insulin sensitivity in skeletal muscle, further support of the mitochondrial ROS in muscle insulin resistance [62]. Thus mitochondrial oxidative stress plays an important role in the initiation of insulin resistance.

Diabetes is linked with multiple cardiovascular complications including accelerated atherosclerosis, augmented ischemic injury post-myocardial infarction, diabetic retinopathy, and nephropathy. In a mouse model of diabetes induced by streptozotocin injection, retina of diabetic mice had two-fold increase in superoxide levels, 40% reduction in GSH levels and 20% reduction in complex III activity, and increased mitochondrial membrane permeability. All these changes were attenuated in mice overexpressing MnSOD, which also experience reduced vascular histopathology, indicating the role of mitochondrial oxidative stress in retinopathy [167]. Cardiac mitochondria in diabetic mice also displayed increased mitochondrial membrane permeability, which has been shown to contribute to the increased propensity for I/R injury in diabetic hearts. Daily intraperitoneal injection of MTP-131 (analogous to SS-31) for 4 days partially reversed increased mPTP opening in diabetic heart mitochondrial. In the same study, Sloan et al. showed that the administration of MTP-131 peptide during reperfusion reduced I/R injury in diabetic hearts, supporting the role of mitochondrial ROS and mPTP opening in I/R injury in diabetic cardiomyopathy [168].

Despite the evidence supporting the role of mitochondrial oxidative stress in experimental models of diabetes, there are mixed results on the protective role of antioxidant treatments in diabetes or its complications from clinical trials [165]. For instance, the HOPE trial showed vitamin E treatment for 4.5 years fails to confer benefit in cardiovascular outcomes and nephropathy [169]. Results of SECURE trial and PPP trial also failed to demonstrate any protective effects with vitamin E treatment [165]. On the other hand, clinical trials on α-lipoic acid have shown more promising results than vitamin E trials. Multiple studies with α-lipoic acid, including ALADIN study, DEKAN study, and SYDNEY trial, have demonstrated its protective effect on diabetic neuropathy [165,170-174].

It is possible that increased mitochondrial ROS may play a larger role in the development of insulin resistance before the onset of chronic hyperglycemia. A recent study using the streptozotocin-induced mouse model of type 1 diabetes actually found reduced mitochondrial function and superoxide production in diabetic kidneys and suggested that this may be due to reduced mitochondrial biogenesis caused by lower PGC1α expression [175]. The investigators postulated that reduced mitochondrial biogenesis led to reduction in activity of AMPK, the master energy sensor. Activation of AMPK restored mitochondrial function and superoxide production, and this was associated with a beneficial reduction in renal pathology. Thus chronic mitochondrial oxidative stress may actually result in reduced mitochondrial function in the later stages of diabetes, and that restoration of mitochondrial structure and function may be necessary to prevent the decline in organ function.

It was recently reported that SS-31 significantly reduced diabetic retinopathy [63]. Daily treatment with SS-31 over 4 months in the rat streptozotocin model significantly prevented the loss of mitochondrial cristae and mitochondrial swelling in retinal epithelial cells. SS-31 also protected the inner blood-retinal barrier, and this was due to preservation of tight junctions in the retinal blood vessels, suggesting adequate ATP production is required to maintain the cytoskeleton of the endothelial cells. Oxidative markers such as 8-OHdG and nitrotyrosine were significantly reduced in the SS-31-treated diabetic animals. The upregulation of VEGFR2 was also significantly attenuated, and this suggests that SS-31 can reduce neovascularization. Interestingly, SS-31 had no effect on blood glucose, but clearly prevented the effects of hyperglycemia on retinal structure and function.

#### Age-related cancer

Mitochondrial ROS leads to oxidative damage in nucleic acids and proteins and has been implicated in carcinogenesis. A recent study demonstrates that loss of mitochondrial cytochrome oxidase is associated with the development of colonic dysplasia (precancerous state) in patients with ulcerative colitis [176]. Direct evidence for the role of mitochondrial ROS in age-related cancer is shown by the effect of mCAT to reduce the non-hematopoietic tumor burden in a mouse end-of-life pathology study [45]. The mCAT expression has also been shown to be protective in an experimental model of metastatic breast cancer (PyMT mice). The mCAT mice displayed reduced invasive grade of primary breast tumor and have 30% less pulmonary metastasis incidence. Both tumor cells and lung fibroblasts in mCAT expressing PyMT mice have reduced intracellular ROS and increased resistance to H_2_O_2_-induced cell death, which may confer the protective effects in mCAT mice [177].

Ataxia telangiectasia mutated (ATM) kinase plays a central role in the DNA-damage response and redox sensing by the phosphorylation of many key proteins that initiate activation of the DNA damage checkpoint, leading to cell cycle arrest, DNA repair, or apoptosis. In addition to severe ataxia due to cerebellar degeneration, ataxia telangiectasia patients also have increased risk of lymphomas and leukemias, as well as immune defect [178]. ATM null mice (ATM^-/-^) develop thymic lymphomas, despite very mild neurodegenerative phenotypes. Reducing mitochondrial ROS by mCAT in ATM^-/-^ mice reduced propensity to develop thymic lymphoma, improved bone marrow hematopoiesis, and macrophage differentiation *in vitro*, and partially rescued memory T-cell development [178].

### Mitochondrial protective strategies as potential therapeutics for aging-related diseases

Meta-analyses of several clinical trials using antioxidant supplement have shown largely disappointing results [13]. With strong evidence of the central role of mitochondrial oxidative stress and damage in several age-related diseases as revealed by the mCAT model, there have been several attempts to develop mitochondria-targeted antioxidants. The most common approach used for delivering compounds into mitochondria have relied on the conjugation of known redox agents to triphenylphosphonium ion (TPP^+^) to take advantage of the potential gradient across the inner mitochondrial membrane. The second major category is aromatic-cationic tetrapeptides that selectively target the inner mitochondrial membrane without relying on mitochondrial potential.

#### TPP^+^ conjugated antioxidants

The mitochondrial inner membrane has a negative potential gradient (-150-180 mV) that is generated as a result of the release of protons from the mitochondrial matrix to the intermembrane space. The negative potential serves as a basis for the use of lipophilic cations to deliver redox agents into the mitochondrial matrix. This method can potentially result in 100- to 1,000-fold accumulation of drugs within the mitochondrial matrix [179]. TPP^+^ has been conjugated to coenzyme Q (MitoQ) and plastoquinone (SkQ1) [30,31]. By preferentially accumulating in the mitochondrial matrix, these TPP^+^-conjugated antioxidants are more potent than their lipophilic counterparts in reducing intracellular ROS, preserving reduced thiols, and reducing oxidative cell death [180,181], This lipophilic cation approach has also been used to generate other mitochondrial-targeted antioxidants to decrease superoxide (MitoSOD), hydrogen peroxide (MitoPeroxidase), ferrous iron (MitoTEMPO), and lipid peroxidation (MitoE2) (see review [182]).

MitoQ had been shown to improve pathology associated with antioxidant deficiency and prolong lifespan of SOD-deficient flies, however, it failed to show lifespan extension in normal WT flies [183]. Indeed, there was a dose-dependent increase in toxicity of MitoQ in flies [183]. MitoQ and SkQ1 have been shown to be effective in reducing ischemia-reperfusion injury [68,184,185]. Dikalova et al. reported that MitoQ treatment for 8 weeks reduced systolic blood pressure and cardiac hypertrophy in spontaneous hypertensive rats [66,186]. The plausible mechanism of blood pressure lowering effect is the improved bioavailability of endothelial nitric oxide. There is evidence that MitoQ can protect against endotoxin-induced cardiac dysfunction [187]. As mentioned earlier, MitoQ was also found to be protective in animal models of neurodegenerative diseases such as AD and PD [164,188]. A series of papers reported that SkQ1 prolonged lifespan, reduced ischemia-reperfusion injury, inhibited tumor development, and returned vision to blind animals [30,67-69].

However, recent reports suggest that MitoQ can actually increase superoxide production at Complex I [189,190] and both MitoQ and SkQ were reported to inhibit mitochondrial bioenergetics [191,192]. Thus while these TPP^+^-conjugated antioxidants can reduce mitochondrial ROS, they may also reduce oxidative phosphorylation and ATP production.

MitoQ has been evaluated in two clinical trials. A small trial of MitoQ in 30 patients with hepatitis C revealed a significant reduction in alanine aminotransferase after 28 days of treatment [193]. However, a double-blind, placebo-controlled trial in patients with PD showed that MitoQ treatment over 12 months did not slow the progression of PD [194]. It is unclear whether clinical development of MitoQ is being continued at this time. On the other hand, SkQ1 eye drops appear to have been approved for dry eye and are available in Russia.

#### SS peptides

The Szeto-Schiller (SS) compounds are tetrapeptides with an alternating aromatic-cationic amino acids motif, which was serendipitously found to preferentially concentrate in the inner mitochondrial membrane greater than 1,000-fold compared with the cytosolic concentration [68,190,195]. Although these peptides carry 3+ net charges, the mitochondrial uptake of these SS peptides is not dependent on mitochondrial potential, as they are also concentrated even in the depolarized mitochondria [190,195]. SS-31 (H-D-Arg-Dmt-Lys-Phe-NH_2_) was originally thought to exert its beneficial effect solely by the free radical scavenging activity of dimethyl tyrosine [66]. SS-31 is able to scavenge H_2_O_2_ hydroxyl radical and peroxynitrite *in vitro* in a dose-dependent manner [195,196].

A recent study revealed that in addition to this ROS scavenging capacity, SS-31 selectively binds to cardiolipin on the inner mitochondrial membrane via both electrostatic and hydrophobic interactions [61]. Cardiolipin is a phospholipid that is uniquely expressed on the inner mitochondrial membrane and plays an important role in the maintenance of cristae structure and formation of super complexes to facilitate electron transfer in the electron transport chain [197-199]. Cardiolipin also plays a role in anchoring cytochrome c to the inner mitochondrial membrane and facilitates electron transfer from complex III to complex IV [200,201]. Although electrostatic interaction with cardiolipin is important for cytochrome c to function as an electron carrier, hydrophobic interaction with cardiolipin tends to cause cytochrome c to unfold and dramatically enhances its peroxidase activity causing cardiolipin peroxidation [202-204]. The oxidation of cardiolipin disturbs cardiolipin microdomains on the inner mitochondrial membrane and causes the loss of cristae curvature and super complex formation. Disruption of super complex formation not only reduces oxidative phosphorylation but also increases ROS formation by complex I [205].

We recently showed that the binding of SS-31 to cardiolipin alters the interaction of cardiolipin with cytochrome c, and favors its electron carrier function while inhibiting peroxidase activity by protecting the Met80-heme ligand [32,206]. By promoting cytochrome c reduction, SS-31 increases electron flux in mitochondria and accelerates ATP production [206]. At the same time, SS-31 inhibits ROS generation and inhibits cytochrome c peroxidase activity, thereby preventing cardiolipin peroxidation and loss of cristae membranes [206]. Thus, SS-31 is a multifunctional mitoprotective compound that acts by promoting bioenergetics, reducing ROS production, scavenging excess ROS, inhibiting cardiolipin peroxidation, and preserving mitochondrial structure.

These unique properties of SS-31 are particularly effective in minimizing ischemia-reperfusion injury. After prolonged ischemia, the hydrophobic interaction between cardiolipin and cytochrome c is enhanced by low ATP concentration [207,208] and this would inhibit mitochondrial respiration at a time when ATP synthesis is necessary for survival. SS-31 is able to increase oxygen consumption and ATP synthesis under these conditions, thus accelerating ATP production upon return of blood flow to minimize cell death and promote organ recovery. By inhibiting cardiolipin peroxidation during reperfusion, SS-31 also preserves mitochondrial cristae and maintains ATP synthesis after ischemia. Numerous preclinical studies support these claims. Studies in models of renal ischemia reperfusion have demonstrated that SS-31 protects mitochondrial cristae architecture and prevents swelling during ischemia and reperfusion [61,209]. This results in more rapid ATP production upon reperfusion and preservation of the cytoskeletal integrity of the epithelial cells, and amelioration of acute kidney injury [61,209].

SS-31 has also been shown to reduce cardiac ischemia reperfusion injury and reperfusion arrhythmia and better preserve myocardial function in various infarct models [59,60,64,196]. SS-31 reduced infarct size in rabbits and sheep after coronary artery ligation, attenuated the extent of no-reflow in rabbits, and reduced infarct size in isolated perfused guinea pig hearts. SS-31 also reduced infarct size in a mouse model of cerebral ischemia and attenuated glutathione depletion when administered at the onset of reperfusion [210].

In addition to ischemia-reperfusion injury, SS-31 has shown impressive effects in preclinical models of heart failure. SS-31 ameliorated Angiotensin-II induced cardiac hypertrophy and diastolic dysfunction, as well as Gαq overexpression-induced heart failure, despite the absence of a blood pressure lowering effect [55]. SS-31 also reduced systolic heart failure in a pressure-overload model of transverse aortic constriction (TAC). Ultrastructural studies confirmed that SS-31 protected cardiac mitochondria in the TAC model and proteomic analyses showed that SS-31 attenuated the majority of the changes in mitochondrial and non-mitochondrial proteins [56]. By protecting mitochondrial function and bioenergetics in the heart, SS-31 prevented myocardial remodeling and fibrosis. The efficacy of SS-31 in combating heart failure has been confirmed in a post-myocardial infarction canine heart failure model, Sabbah et al. demonstrates that short-term administration of Bendavia for 2 h significantly increased ejection fraction, stroke volume, cardiac output, and LV contractility index (dP/dt) [57]. These findings suggest that the improvement of LV function is likely the result of improved cardiac energetics. Long-term administration for 3 months significantly improved ejection fraction and reduced LV end-diastolic pressure [58].

SS-31 has also been shown to be beneficial in many other models of age-associated diseases, including PD [65], AD [152], skeletal muscle aging, disuse skeletal muscle atrophy [210], [54] insulin resistance [62], and diabetic complications. Some of these studies were mentioned in the previous sections (see section Mitochondrial oxidative stress in healthspan above), and an extensive review of these studies was published recently [32].

Given the very promising preclinical efficacy data, SS-31 entered into clinical trials using a clinical formulation named Bendavia [32]. Several Phase I studies have assessed the safety, tolerability and pharmacokinetics of Bendavia in healthy male and female human subjects with intravenous and oral dosing. The highly predictable pharmacokinetics and safety profile of Bendavia have led to Phase II trials in patients. The first multinational phase II study is focused on cardiac ischemia-reperfusion injury for patients experiencing ST-elevation myocardial infarction [211]. A second ongoing phase II trial is for treatment of acute kidney injury in hypertension. A third phase II trial is planned for the treatment of congestive heart failure.

These clinical studies are generally designed to address the efficacy of SS-31 in the treatment of age-associated cardiorenal diseases. It will eventually be important to also establish whether these mitochondria-targeted antioxidants can delay aging and other age-related degenerative diseases.

## Conclusion

Substantial evidence supports the central role of mitochondrial oxidative stress in aging and healthspan. Despite the disappointing outcomes of non-targeted antioxidants in clinical trials, there is growing evidence for the beneficial effects of mitochondrial-targeted antioxidants in aging and age-related diseases. Genetic and pharmacological approaches reducing mitochondrial oxidative stress (either by direct antioxidant or indirectly through preservation of mitochondrial structure and function) attenuate the phenotypes of cardiac aging, age-related cardiovascular diseases, skeletal muscle aging, neurodegenerative diseases, diabetes, and cancer various animal models (summarized in Table 1). Moreover, based on promising preliminary results in small and large mammals, mitochondrial-targeted antioxidants have moved into clinical trials. Further studies are necessary to investigate many of the remaining questions in this field, while examining the potential application of mitochondrial targeted therapeutics in the treatment or prevention of specific diseases as well as improved healthspan in general.

## Competing interests

HH Szeto is the inventor of SS-31 and the Cornell Research Foundation (CRF) holds several patents covering the SS peptides and a patent application has been filed for the findings described in this article, with HH Szeto, PS Rabinovitch and DF Dai as inventors. CRF has licensed the SS peptide technology for further research and development to a commercial enterprise in which CRF and HH Szeto have financial interests.

## Authors’ contributions

DFD and YAC drafted and revised the manuscript. DM helped to draft and made critical revision to the manuscript. HHS and PSR made critical revision to the manuscript. All authors read and approved the final manuscript.
